# Euterpe Oleracea Martius (Açaí) Extract and Resistance Exercise Modulate Cardiac Parameters of Hypertensive Rats

**DOI:** 10.3390/life14091101

**Published:** 2024-09-02

**Authors:** Pilar Barbosa de Meireles, Denise Coutinho de Miranda, Anselmo Gomes de Moura, Willian Cruz Ribeiro, Ângela Quinelato Oliveira, Luciano Bernardes Leite, Pedro Forte, Lúcia Ribeiro, Samuel G. Encarnação, Luiz Otávio Guimarães-Ervilha, Mariana Machado-Neves, Mariana Moura e Dias, Iasmim Xisto Campos, Emily Correna Carlo Reis, Maria do Carmo Gouveia Peluzio, Antônio José Natali, Victor Neiva Lavorato

**Affiliations:** 1 Department of Physical Education and Nutrition, Governador Ozanam Coelho University Center, Ubá 36506-022, MG, Brazil; pimeireless@gmail.com (P.B.d.M.); denise.miranda@unifagoc.edu.br (D.C.d.M.); anselmo.moura@unifagoc.edu.br (A.G.d.M.); wzenaide29@gmail.com (W.C.R.); angela.quinelato.o@gmail.com (Â.Q.O.); victor.lavorato@unifagoc.edu.br (V.N.L.); 2 Laboratory of Exercise Biology, Federal University of Viçosa, Viçosa 36590-000, MG, Brazil; luciano.leite@ufv.br (L.B.L.); anatali@ufv.br (A.J.N.); 3 Department of Sports, Instituto Politécnico de Bragança, 5300-253 Bragança, Portugal; 4 Department of Sports, Higher Institute of Educational Sciences of the Douro, 4560-708 Penafiel, Portugal; 5 CI-ISCE, ISCE Douro, 4560-547 Penafiel, Portugal; 6 Research Center for Active Living and Wellbeing (LiveWell), Instituto Politécnico de Bragança, 5300-253 Bragança, Portugal; 7 Centro de Investigação de Montanha (CIMO), Instituto Politécnico de Bragança, Alameda Santa Apolónia, 5300-253 Bragança, Portugal; luciamarisa.maiararibeiro@uvigo.es; 8 Laboratório Associado para a Sustentabilidade e Tecnologia em Regiões de Montanha (SusTEC), Instituto Politécnico de Bragança, Campus de Santa Apolónia, 5300-253 Bragança, Portugal; 9 Facultade de Ciencias, Universidad de Vigo, 32004 Ourense, Spain; 10 Department of Physical Education, Sport and Human Movement, Universidad Autónoma de Madrid (UAM), Ciudad Universitaria de Cantoblanco, 28049 Madrid, Spain; 11 Departament of Biological Sciences, Federal University of Viçosa, Viçosa 36590-000, MG, Brazil; luiz.ervilha@ufv.br (L.O.G.-E.);; 12 Department of Nutrition and Health, Federal University of Viçosa, Viçosa 36590-000, MG, Brazilmpeluzio@ufv.br (M.d.C.G.P.); 13 Department of Veterinary Medicine, Federal University of Viçosa, Viçosa 36590-000, MG, Brazil; emily.carlo@ufv.br

**Keywords:** heart, resistance exercise, açai, hypertension

## Abstract

Background: The study evaluated the effects of resistance exercise training and açaí supplementation on cardiac parameters in hypertensive animals. Methods: For this study, rats from the Wistar and SHR lines (spontaneously hypertensive rats) were used. The animals were divided into 5 groups: Wistar Control (C); Control Hypertensive (H); Trained Hypertensive (HT); Hypertensive and Supplemented with Açaí (HA); and Hypertensive Trained and Supplemented with Açaí (HAT). Resistance exercise training was carried out through climbing. The supplemented groups received 3 g of açaí/kg of body mass. The animals’ systolic blood pressure (SBP), body mass, and physical test were measured at the beginning and end of the intervention. At the end, an echocardiographic analysis was performed. Histological analysis and oxidative stress of the LV were performed. Results: It was found that hypertensive animals showed an increase in SBP, and the treatments reduced this parameter. The trained groups achieved higher values of maximum carrying load. Hypertension increased the dimension of the left ventricular free wall in diastole and reduced ejection and shortening fractions. The trained groups showed improvement in ejection and shortening fractions. The H group increased the proportion of extracellular matrix and reduced the proportion of cells, with the HAT group attenuating this change. Cell diameter was greater in group H, and all treatments reduced this parameter. Hypertension increased the concentration of malondialdehyde and decreased catalase activity in LV. The treatments managed to mitigate this damage. Conclusions: It is concluded that the treatments managed to generate positive cardiovascular adaptations, and their combination enhanced these effects.

## 1. Introduction

Systemic arterial hypertension (SAH) is a major public health problem and increases the risk of developing cardiovascular and cerebrovascular diseases [1]. To be considered hypertensive, the individual must have blood pressure levels equal to or greater than 130/80 mmHg [2]. High levels of pressure in the blood vessels cause the heart to suffer greater overload to correctly distribute blood throughout the body, which can cause consequences such as acute myocardial infarction, kidney failure, and brain stroke [3].

Animal research involving the study of SAH uses a variety of experimental models, with the SHR (spontaneously hypertensive rat) being one of the most used strains. These animals are similar in comparison to the development of SAH in humans [4].

To treat the effects imposed by SAH, therapeutic alternatives have been applied, such as supplementation with Euterpe oleracea Mart (açaí) extract. The fruit, popularly cultivated on a large scale in the Amazon region, is a perishable food and has a high lipid content and many phenolic compounds in its composition, which gives it great antioxidant capacity [5]. Among the phenolic compounds, anthocyanins stand out, which contribute to the purple color of the fruit and are responsible for combating reactive oxygen species. These, when not eliminated correctly, can lead to increased oxidative stress, and contribute to the emergence of chronic noncommunicable diseases, such as hypertension [6].

Another nonpharmacological treatment for the effects of SAH is physical training. Resistance exercise training is one of the physical activities that obtain positive results related to hypertension, as it reduces systolic and diastolic pressure [7]. It was found that a resistance exercise training program can generate adaptations in the heart, with an increase in the ejection fraction and shortening fraction, in addition to improving local microcirculation [8]. Another study showed that 12 weeks of resistance exercise training reduced oxidative stress in the hearts of SHR animals [9].

However, there are still no conclusive studies of the association of physical resistance exercise training and açaí supplementation in relation to their benefits on cardiac parameters in hypertensive rats. Therefore, the aim of the present study was to evaluate the effects of resistance exercise training and açaí supplementation on cardiac parameters in hypertensive animals.

## 2. Materials and Methods

### 2.1. Animals

Wistar and SHR rats (~60 days old) were used in the present study. All the experiments were approved by the Ethics Committee for Animal Use at the Governador Ozanam Coelho University Center (02/2022).

The rats were kept in cages under a 12/12 h light/dark regime in a controlled temperature (22 °C) room, had water and chow ad libitum, and were observed daily. The body mass of the animals was obtained weekly. For the analyses, initial and final body mass were used. The rats were divided into five experimental groups, namely: Wistar Control (C, n = 10); Control Hypertensive (H, n = 10); Trained Hypertensive (HT, n = 10); Hypertensive and Supplemented with Açaí (HA, n = 10); and Hypertensive Trained and Supplemented with Açaí (HAT, n = 10). The treatments lasted 12 weeks (5 times a week).

### 2.2. Acquisition, Preparation, and Administration of Açai Pulp

The tests were conducted using pasteurized açaí pulp obtained from Layne Agroin-dústria Ltd. (Guidoval, Minas Gerais, Brazil). This commercial pulp was produced by macerating the açaí pericarp and mixing it with water. Açaí pulps from the same manufacturing batch, sourced during the same week as the animals’ arrival at the laboratory, were used. The pulp was stored at −20 °C until use, when it was thawed and filtered through Whatman No. 1 filter paper (Maidstone, UK). The resulting aqueous açaí extract was administered directly to the animals and used for proximate, phytochemical, and antioxidant characterization. The groups supplemented with açaí received a daily dose of 3 g of açaí/kg of body mass through gavage [10].

### 2.3. Centesimal Composition of Açai

The analysis of the centesimal composition of the filtered açaí pulp was carried out in accordance with the analytical standards of the Adolfo Lutz Institute. Moisture determination was carried out using the oven drying method at 105 °C, until a constant weight was obtained. Lipids were extracted by the Bligh and Dyer method due to the large water content that is normally found in the sample, using chloroform and methanol as solvents. Ash was determined after incineration of the material in a muffle furnace at 550–660 °C. Protein determination was based on the Kjeldahl method, in which the amount of protein was calculated by the product of the amount of total nitrogen, considering 6.25 as the conversion factor. Carbohydrates were determined by the difference in relation to the other quantified components.

### 2.4. Antioxidant Capacity

The determination of the antioxidant capacity of the filtered açai pulp was carried out using 2,2-diphenyl-1picrylhydrazyl (DPPH). In test tubes, 3.9 mL of 60 μmol/L DPPH solution dissolved in 80% methanol and 0.1 mL of açaí pulp were added. After homogenization, the mixtures remained at rest for 30 min in the absence of light. The control preparation was carried out following the same procedures with distilled water replacing the sample. The absorbances were read at 515 nm using a spectrophotometer (Biospectro^®^—São Paulo (SP), Brazil), and 80% methanol was used as a blank. A standard curve was constructed using 6-hydroxy-2,5,7,8-tetramethylchrome-2-carboxylic acid (Trolox) (Sigma-Aldrich^®^—St. Louis, MO, USA) as a reference antioxidant in solutions with concentrations ranging from 100 to 800 μmol./L. The antioxidant capacity of Trolox equivalents (TEAC) was expressed as μmol/L of TEAC/g of açai.

### 2.5. Systolic Blood Pressure

Systolic blood pressure (SBP) measurements were performed using a noninvasive method using adapted tail plethysmography. During measurements, the animals were positioned in the containment apparatus and heated passively to achieve vasodilation of the caudal artery through exposure to a temperature of 29–32 °C for 10 min. Then, a pressure cuff was placed 3 cm from the tip of the tail. After capturing the pulse, the cuff was inflated to obtain SBP measurements.

### 2.6. Physical Test and Resistance Exercise Training Protocol

The Hornberger and Farrar [11] protocol was adapted to the needs and execution of the research. The rats were familiarized for two weeks (three times a week) with the practice of resistance training, which consists of climbing a vertical ladder (1.1 × 0.18 m, 2 cm spacing between the steps, 80° inclination) with an external overload fixed to the tail. At the end of the climb, they rested for 120 s. This procedure was repeated until the rats voluntarily climbed the ladder three consecutive times, without stimulation. In the second week of adaptation, the rats voluntarily climbed the ladder in two to three series, with a 20 g apparatus attached to the proximal part of the tail using adhesive tape.

After the period of familiarization with the ladder, a maximal carrying load test was performed, as previously described [12]. At the end of each month of resistance training, this test was performed again with the aim of readjusting the resistance training load; and at the end of the last week of resistance training, the animals’ strength gain was verified. The maximal carrying load was used as an index of tolerance to physical effort.

After the load test, resistance physical training began. The training was divided into three mesocycles, starting with 5 sets and 60% of the maximum load and progressing to 12 series and 75% of the maximal carrying load [13].

### 2.7. Echocardiographic Analysis

The animals were immobilized using isoflurane anesthesia. The anesthetic was administered through a vaporizer, and induction was performed by administering 3% isoflurane and 100% O_2_ at a constant flow of one liter per minute, for a period of approximately 3 min. Isoflurane was maintained at 1.5% through a small nose cone (adapted for rats) for a period of 10 to 12 min to perform echocardiography. The exam includes two-dimensional (2D) studies with a fast-sampling rate (frame rate) of 120 fps and M-mode, using the ultrasound system (MyLabTM30—Esaote, Italy) with an 11.0 MHz phased array transducer nominal frequency [14]. The thickness of the posterior wall of the left ventricle (LV) at the end of diastole and at the end of systole, the diastolic and systolic thickness of the interventricular septum, and in addition the diastolic dimensions of the right ventricle and systolic and diastolic dimensions of the LV were measured using a modified method recommended by the Society American Echocardiography during three consecutive cardiac cycles [15]. From the previous analyses, ejection fraction and shortening fraction were calculated.

### 2.8. Histological Analysis of the Heart

After euthanasia by decapitation, the heart was removed after sectioning the ascending aorta. The LV was separated, and fragments of this tissue were fixed for 48 h in Carlsson formalin (10%). Subsequently, the fragments were dehydrated in 80%, 90%, 95%, and absolute ethyl alcohol for 30 min each, and kept in the resin for 24 h. After 24 h, the samples were embedded in the resin with hardener and stored in an oven at 60 °C for 48 h. The samples were then sectioned at 5 µm thickness using a rotating microtome (Spencer, model 19459, Fresno, CA, USA) and stained with hematoxylin and eosin. The slides were viewed and images were captured using a light microscope (Olympus BX-50, Tokio, Japan) connected to a digital camera (Olympus Q Color-3, Tokio, Japan).

To quantify the proportions of the extracellular matrix and the cardiomyocytes, 10 random images were used for each animal. A grid with 266 intersections was superimposed on each image. The intersections in the cardiomyocytes and the extracellular matrix were counted, and then the percentage was calculated. Furthermore, the diameter of the cardiomyocytes was also evaluated through interactive measurements, by drawing a straight line connecting the upper and lower cellular limits in the nucleus region. Only fibers with visible nuclei and cell boundaries were included, with a total of 100 cardiomyocytes analyzed for each animal. All the measurements were performed using Image-Pro Plus 4.5 software (Media Cybernetics, Rockville, MD, USA).

### 2.9. Analysis of Oxidative Stress

Fragments of LV (150 mg) were homogenized in 1.5 mL of phosphate buffer containing EDTA and centrifuged at 12,000 rpm, for 10 min, at 4 °C. The supernatant and pellet were used for the following biochemical analyzes. Catalase activity was assessed according to the method described by Aebi [16], measuring the rate of decomposition of hydrogen peroxide (H_2_O_2_). Superoxide dismutase (SOD) activity was determined by a method adapted from Dietrich et al. [17]. Furthermore, the determination of lipid peroxidation in cardiac homogenates was performed by detecting malondialdehyde (MDA) [18]. Reduced glutathione (GSH) content was determined as proposed by Griffith [19]. The protein concentration in the LV homogenates was measured by the method of Lowry et al. [20].

### 2.10. Statistical Analysis

Initially, the Kolmogorov–Smirnov normality test was performed. When the data distribution was considered normal, one-way ANOVA was performed, with Tukey’s post hoc to locate the difference between the groups. When the distribution was considered non-normal, the Kruskal–Wallis test was performed, with Dunn’s post hoc. To assess whether there was a difference between initial and final body mass, between initial and final physical capacity, and between initial and final SBP, the Mixed ANOVA was used considering the factor group (C, H, HA, HT, HAT) for between-subject interactions and the factor time (pre and post) for within-subject interactions. The Shapiro–Wilk test checked the data normality. The assumptions of homogeneity of the variances and sphericity were, respectively, verified with the Levene’s and Mauchly’s tests. If the sphericity assumption was broken, the statistical calculation considered the Greenhouse–Geisser correction for the *p*-value. If any statistically significant difference was found, the Bonferroni’s post hoc test was applied to make paired comparison between groups over the moments. The Eta squared showed (η^2^) and was calculated to measure the effect size of the significant effects and interactions, considering the cutoffs of ≤0.10 = small, ≥0.25 = medium, and ≥0.40 = large according to Cohen’s guidelines. The results were expressed as mean ± standard deviation of the mean. The significance level adopted was *p* < 0.05 for all the statistical analyses. All the analyses were performed using GraphPad Prism 8.1^®^ software.

## 3. Results

The centesimal composition of the açaí pulp presented 93.53 ± 1.46% moisture. The dry weight composition, calculation of phenolic compounds, and antioxidant capacity of the açaí are observed in Table 1.

Table 2 presents data regarding body mass, SBP, and maximum carrying load at the beginning and end of the intervention period. There was a significant effect of group, F(4, 48) = 23.13; *p* < 0.05, η^2^ = 0.61, and time, F(1, 48) = 177.25; *p* < 0.05, η^2^ = 0.40 on the body mass. However, there were no group x time interactions F(4, 48) = 1.89; *p* > 0.05, η^2^ = 0.03, suggesting that all the groups changed similarly over time. The within-subjects comparisons showed that body mass increased in all groups after the intervention, C (*p* = 0.03), H, HA, HAT (*p* < 0.0001), HT (*p* < 0.001). The between-comparison showed that the C group had higher body mass than all the groups in the pre-test and post-test, *p* < 0.0001.

Regarding SAP, there was a significant effect of group F(4, 48) = 198.63; *p* < 0.05, η^2^ = 0.86, time F(1, 48) = 25.06; *p* < 0.05, η^2^ = 0.24. Therefore, there were significant group x time interactions F(4, 48) = 9.40; *p* < 0.05, η^2^ = 0.32, where the within-comparisons revealed that there were significant reductions in SAP only in the groups HA (*p* < 0.001), HAT (*p* < 0.0001), and HT (*p* < 0.0001), with no changes in H and C over time (*p* > 0.05). The between- subjects comparison showed that C presented lower SAP than all the groups in the pre- and post-test (*p* < 0.05) and the H groups presented higher SAP than all the groups in the post-test (*p* < 0.05).

Finally, the analysis of the maximum carrying load also showed a significant effect of group F(4, 48) = 21.84; *p* < 0.05, η^2^ = 0.53, time F(1, 48) = 124.80; *p* < 0.05, η^2^ = 0.49. Furthermore, there was also a significant group x time interaction F(3, 48) = 52.97; *p* < 0.05, η^2^ = 0.62, where the within-subjects comparisons identified that only the groups HA (*p* < 0.05), HAT, and HT (*p* < 0.0001) increased the total load carried, whereas the H and C groups did not change the muscle strength (*p* < 0.05). The between-subjects comparisons showed that group H carried less load than HAT and HT in the post-test (*p* < 0.0001) and the HA also presented lower load-carrying capacity than HAT and HT (*p* < 0.0001). The comparisons between H-HA and HAT-HT in the post-test did not show any significant differences in maximum carrying load (*p* < 0.05).

The group HT had greater ventricular mass compared to group C. The heart/final body mass and LV/final body mass ratios were higher in group HT compared to group C. The ventricles/final body mass ratio was higher in all the hypertensive groups compared to group C (Table 3).

Table 4 presents data relating to the echocardiographic examination of the experimental animals. Hypertension increased left ventricular free wall dimension in systole (LVFW-s) and reduced ejection (EF) and shortening (FS) fractions. The HT and HAT groups had increased EF and FS compared to the H group.

**Figure 1:**
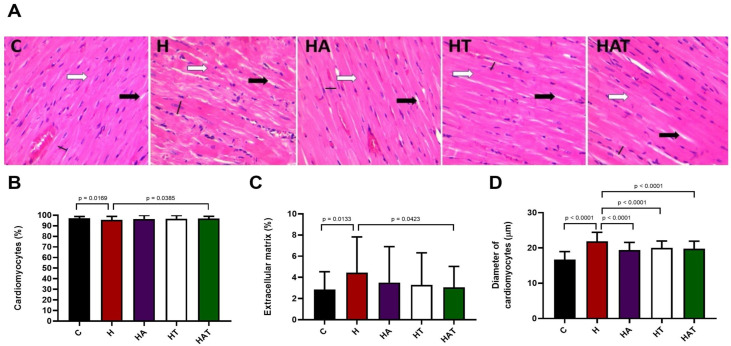
(**A**) Histological photomicrographs of the left ventricle of experimental animals. White arrow: indicates area of cardiomyocytes. Black arrow: indicates extracellular matrix area. Black line: indicates the cell dimension measurement. (**B**) Proportion of cardiomyocytes. (**C**) Proportion of extracellular matrix. (**D**) Diameter of cardiomyocytes. Data expressed as mean ± standard deviation of 6 to 8 animals per experimental group. Wistar Control (C); Control Hypertensive (H); Trained Hypertensive (HT); Hypertensive and Supplemented with Açaí (HA) and Hypertensive Trained and Supplemented with Açaí (HAT).

Figure 1 shows the histological data of the hearts. Hypertension increased the percentage of extracellular matrix and reduced the percentage of cardiomyocytes. The treatments together led to the reduction of these parameters. Hypertension increased the size of the cardiomyocytes, and all the treatments were able to attenuate this increase.

**Figure 2:**
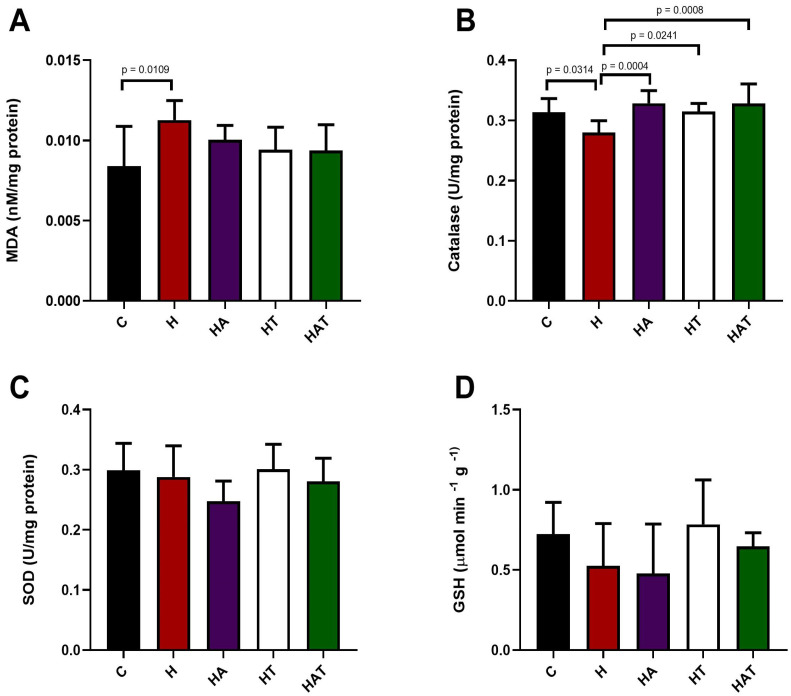
(**A**) Oxidative markers and activity of antioxidant enzymes in the left ventricle of experimental animals. A: Malondialdehyde (MDA) concentration. (**B**) Catalase levels. (**C**) superoxide dismutase (SOD) levels. (**D**) Reduced glutathione (GSH) levels. Data expressed as mean ± standard deviation of 6 to 8 animals per experimental group. Wistar Control (C); Control Hypertensive (H); Trained Hypertensive (HT); Hypertensive and Supplemented with Açai (HA) and Hypertensive Trained and Supplemented with Açai (HAT).

Figure 2 presents the data referring to the analyses of LV oxidative stress. Group H had a higher concentration of MDA compared to group C. Furthermore, Group H showed lower catalase levels compared to the other experimental groups.

## 4. Discussion

It is well established that hypertension causes remodeling of the heart and specifically of the LV. High blood pressure levels increase the demand on the LV, generating adaptations that allow the deposition of new sarcomeres in parallel, and lead to concentric hypertrophy [21]. In fact, an increase in the ventricles/body mass ratio, LVFW-s, and cardiomyocyte diameter was observed in group H, indicating increased hypertrophy in the hearts of these animals.

Hypertension is associated with increased oxidative stress, local inflammation, and collagen deposition, causing interstitial fibrosis [22]. Fibrosis increases myocardial stiffness and, consequently, increases the risk of cardiac dysfunction in hypertension. Our results show that hypertension increased MDA concentration and reduced catalase activity. Furthermore, hypertension reduced the proportion of cardiomyocytes and caused an increase in the extracellular matrix, which suggests greater formation of fibrosis. An increase in collagen content in the LV was observed eight weeks after the establishment of hypertension [23].

In addition to structural changes, it is well documented that hypertension causes electrical and mechanical abnormalities in the heart [24,25]. In this sense, it was seen that hypertension caused a reduction in ejection fraction and shortening fraction.

Regarding the positive effects found through açaí treatment, a reduction in SBP levels was noted at the end of the intervention period. Corroborating our findings, the study by Costa et al. [26] showed that animals with renovascular hypertension that were treated with açaí through water consumed had reduced SBP levels after six weeks of intervention. Açaí has many polyphenols, mainly anthocyanins, which gives it great antioxidant capacity [27]. It has been shown that regular consumption of anthocyanins and flavonoids is correlated with improved blood pressure levels [28].

Açaí consumption can improve oxidative stress in a heart affected by SAH, neutralizing reactive oxygen species by increasing the action of antioxidant enzymes and, consequently, reducing the inflammatory process and collagen deposition, preventing changes in cardiac compliance [26,29]. In fact, our study showed that the HA group showed increased catalase activity; the increased MDA concentration in LV was attenuated. However, the present study did not observe a reduction in the proportion of extracellular matrix in the group of hypertensive patients treated with açaí. The study by Figueiredo et al. [30] identified that the hearts of infarcted rats treated with 2 or 5% açaí in the diet showed lower activity of the antioxidant enzyme SOD [31]. Furthermore, infarcted animals treated with açaí showed an improvement in the inflammatory state of the heart, via an increase in the concentration of interleukin 10 [30].

Açaí treatment reduced the diameter of the cardiomyocytes. It was found that açaí supplementation increases the aortic lumen in hypertensive animals [32]. These data indicate that a possible improvement in the afterload in animals treated with açaí may have generated adaptations to reduce sarcomere growth in parallel and, consequently, smaller diameter. In line with our findings, an extract rich in polyphenols from grapes reduced the cross-sectional area of cardiomyocytes from hypertensive rats [33].

Resistance exercise training reduced SBP and increased ventricular mass and the ratios of the heart, ventricles, and LV to the body mass. Furthermore, resistance exercise training reduced the diameter of the cardiomyocytes. The mechanisms by which physical activity influences blood pressure are complex. A possible explanation involves the improvement of endothelial function and the reduction of peripheral vascular resistance [34]. Lino et al. [35], using the same training program, but for 13 weeks, identified a reduction in SBP and type I and III collagen content in the animals’ aorta. This data may explain the reduction in the diameter of cardiomyocytes in trained rats, leading to a decrease in concentric hypertrophy.

On the other hand, resistance exercise training failed to increase the proportion of cardiomyocytes and reduce the proportion of the extracellular matrix. In addition, resistance exercise training attenuated MDA concentrations and increased catalase activity in cardiac tissue, in relation to the hypertensive group. Despite this, it is known that exercise can have a protective effect on the heart. Resistance exercise training carried out for 12 weeks was able to reduce oxidative stress in the hearts of hypertensive rats, with an increase in SOD and catalase activity [9]. Added to this, Perilhão et al. [13] found that animals that performed resistance exercise training had increased physical capacity, reduced blood pressure, heart rate, isovolumetric relaxation time, and collagen content in the heart, with increased cardiac function, without changes in mass and in the nuclear volume of the cardiomyocytes. Furthermore, Neves et al. [36] analyzed the effect of 12 weeks of resistance training in SHR animals, observing a reduction in the nuclear number of cardiomyocytes. This action is directly linked to stimuli from the renin angiotensin aldosterone system and the sympathetic nervous system [37].

The association of treatments between açaí supplementation and resistance physical training reduced SBP and attenuated the increase in the proportion of extracellular matrix and the diameter of the cardiomyocytes, in addition to attenuating the increase in MDA concentration and increasing catalase activity in the cardiac tissue. Therefore, treatments together seem to enhance isolated effects. In the study by De Bem et al. [37] it was found that the use of açaí associated with aerobic exercise also showed synergy, increasing the expression of proteins involved in skeletal muscle hypertrophy (pAKT and pAMPK). The systemic effects provided by physical exercise and açaí appear, especially in relation to redox modulation, to have enhanced effects on cardiac tissue.

The study has limitations. One of these is the failure to measure diastolic blood pressure, depending on the device used. Another limitation is the absence of molecular analyses that could reinforce or explain the findings of the present study.

## 5. Conclusions

It is concluded that açaí supplementation and resistance exercise training can generate positive adaptations in relation to hemodynamic, functional, and morphometric parameters, in addition to oxidative stress in the cardiac tissue of hypertensive animals. The treatments together were able to enhance the isolated effects.

## Figures and Tables

**Table 1:** Centesimal composition, phenolic compounds, and antioxidant capacity of açaí.

Compounds	Values
Proteins (%)	12.68 ± 1.27
Lipids (%)	50.70 ± 0.28
Fibers (%)	21.47 ± 1.12
Ashes (%)	3.78 ± 0.02
Carbohydrates (%)	11.37
Phenolics (mg GAE/g)	8.02 ± 0.11
Antioxidant capacity (µM Trolox/g)	18.19 ± 0.90

Values are expressed as mean ± standard deviation. Carbohydrates obtained by difference in relation to the other components. GAE, gallic acid equivalent.

**Table 2:** Body mass, systolic blood pressure, and maximum carrying load in the experimental groups.

	C	H	HA	HT	HAT
Initial Body Mass (g)	403.1 ± 41.82 ^Ω^****	289.5 ± 11.67	303.7 ± 19.74	302.2 ± 38.50	294.8 ± 27.50
Final Body Mass (g)	432.7 ± 43.06 ^#^***^Ω^****	349.4 ± 26.12 ^#^****	355.1 ± 30.96 ^#^****	349.9 ± 37.79 ^#^***	348.6 ± 18.51 ^#^****
Initial SBP (mmHg)	116.1 ± 12.44 ^Ω^****	199.0 ± 17.29	197.5 ± 11.97	199.5 ± 10.92	200.0 ± 10.95
Final SBP (mmHg)	117.0 ± 4.07 ^Ω^****	209.4 ± 12.04 ^Ω^****	180.2 ± 13.83 ^#^***	171.4 ± 14.12 ^#^***	166.2 ± 14.20 ^#^***
Initial MCL (g)	361.5 ± 55.99	316.3 ± 51.28	347.4 ± 49.79	325.5 ± 44.29	306.8 ± 33.58
Final MCL (g)	379.5 ± 54.24	344.2 ± 96.05 ^†^****	295.3 ± 41.22 ^#^*^§^****	584.4 ± 34.03 ^#^****	533.8 ± 32.32 ^#^****

Data expressed as mean ± standard deviation of 8 to 10 animals per experimental group. SBP, systolic blood pressure. MCL, maximum carrying load. ^#^, difference to the initial moment within the same group. ^Ω^, difference to the other groups. ^†^, difference from HAT and HT, ^§^, difference from HAT and HT. *, *p* < 0.05, ***, *p* < 0.001, ****, *p* < 0.0001. Wistar Control (C); Control Hypertensive (H); Trained Hypertensive (HT); Hypertensive and Supplemented with Açaí (HA) and Hypertensive Trained and Supplemented with Açaí (HAT).

**Table 3:** Effect of treatments with açaí supplementation and/or resistance exercise training on heart mass and body tissues.

	C	H	HA	HT	HAT	*p*
Heart mass (g)	1.535 ± 0.4090	1.541 ± 0.1142	1.559 ± 0.1355	1.753 ± 0.2794	1.562 ± 0.1356	0.2519
Ventricle mass (g)	0.966 ± 0.1476	1.102 ± 0.1402	1.144 ± 0.0846	1.183 ± 0.2054 *	1.100 ± 0.1047	0.0267
LV mass (g)	0.583 ± 0.3927	0.774 ± 0.1389	0.795 ± 0.0639	0.840 ± 0.1587	0.794 ± 0.0512	0.0666
Heart/Body mass (g)	0.003 ± 0.0012	0.004 ± 0.0003	0.004 ± 0.0003	0.005 ± 0.0013 *	0.004 ± 0.0002	0.0093
Ventricle/Body mass (g)	0.002 ± 0.0003 ^Ω^	0.0031 ± 0.0003	0.0031 ± 0.0002	0.0034 ± 0.0008	0.0031 ± 0.0002	<0.0001
LV/Body mass (g)	0.001 ± 0.0005	0.0022 ± 0.0004	0.0021 ± 0.0001	0.0024 ± 0.0006 *	0.0022 ± 0.0001	0.0276

Data expressed as mean ± standard deviation of 8 to 10 animals per experimental group. LV, left ventricle. *, difference for C group. ^Ω^, difference for the other groups. Wistar Control (C); Control Hypertensive (H); Trained Hypertensive (HT); Hypertensive and Supplemented with Açaí (HA) and Hypertensive Trained and Supplemented with Açaí (HAT).

**Table 4:** Echocardiographic evaluation of the experimental groups.

	C	H	HA	HT	HAT	*p*
RVID-d (mm)	1.60 ± 0.49	2.08 ± 0.43	1.73 ± 0.39	2.01 ± 0.38	2.03 ± 0.57	0.3057
IVS-d (mm)	1.83 ± 0.35	2.08 ± 0.24	2.06 ± 0.31	2.21 ± 0.29	2.15 ± 0.41	0.3555
LVID-d (mm)	7.80 ± 1.24	7.88 ± 0.42	7.71 ± 0.77	7.75 ± 1.20	7.58 ± 0.87	0.9874
LVFW-d (mm)	1.70 ± 0.40	2.16 ± 0.30	2.20 ± 0.20	2.13 ± 0.52	2.31 ± 0.50	0.1242
IVS-s (mm)	2.81 ± 0.54	2.91 ± 1.03	2.38 ± 0.34	2.66 ± 0.28	2.46 ± 0.30	0.4631
LVID-s (mm)	4.68 ± 0.77	5.68 ± 0.33	5.25 ± 0.61	5.20 ± 0.93	5.05 ± 0.49	0.1654
LVFW-s (mm)	2.48 ± 0.26	2.85 ± 0.23 *	2.70 ± 0.18	2.81 ± 0.14	2.78 ± 0.16	0.0328
EF (%)	76.00 ± 2.82	59.67 ± 2.06 *	65.67 ± 4.63	67.33 ± 4.54 ^ᴪ^	67.50 ± 3.27 ^ᴪ^	<0.0001
FS (%)	39.83 ± 2.71	27.83 ± 1.47 *	32.00 ± 3.28	32.83 ± 3.43 ^ᴪ^	32.83 ± 2.63 ^ᴪ^	<0.0001

Data expressed as mean ± standard deviation of 6 animals per experimental group. RVID-d, right ventricular internal dimension in diastole. LVID-d, left ventricular internal dimension in diastole. LVID-s, left ventricular internal dimension in systole. LVFW-s, left ventricular free wall dimension in diastole. LVFW-s, left ventricular free wall dimension in systole. IVS-d, thickness of the interventricular septum in diastole. IVS-s, thickness of the interventricular septum in systole. *, difference for C group. ^ᴪ^, difference for H group. Wistar Control (C); Control Hypertensive (H); Trained Hypertensive (HT); Hypertensive and Supplemented with Açaí (HA) and Hypertensive Trained and Supplemented with Açaí (HAT).

## Data Availability

The data will be shared on reasonable request to the corresponding author.
